# Not Just a Missing Number: A dementia and mental health cohort focused around LGBTQIA+ communities

**DOI:** 10.1002/alz70860_105385

**Published:** 2025-12-23

**Authors:** Sarah Bauermeister

**Affiliations:** ^1^ University of Oxford, Oxford, Oxfordshire, United Kingdom

## Abstract

**Background:**

Members of the LGBTQIA+ community endure many forms of stigma and discrimination and in research, they are frequently ‘non‐identified’. A contributing factor is the historic lack of categorization for non‐binary (male/female) gender identity. This is particularly so for the Trans community who are already marginalized both in society and medical care. The consequence is that the LGBTQIA+ community are thereby excluded by default from important neurodegenerative disease and mental health research because the lack of non‐binary categorization means that members do not/cannot/do not wish to identify with the gender categories provided and are forced to select 'other', 'non‐specified’ or ‘do not wish to answer’. The subsequent result of this in research means that members of the community may be simply recoded as a 'missing number', resulting in the exclusion of their data from important findings, treatments or solutions for neurodegenerative and mental health diseases. The overall objective of ‘Not Just a Missing Number’ (*N*‐Jam) is to address key overlooked outcomes of lack of appropriate categorisation provided for the LGBTQIA+ communities. Importantly, the project will focus on exposures and outcomes relevant to the LGBTQIA+ communities, e.g., social isolation, marginalisation, discrimination, gender specific health misidentification for the Trans community.

**Method:**

Establish personal and public involvement (PPI) and steering groups from within and among the LGBTQIA+ communities

Highlight the current situation and consequences of inadequate LGBTQIA+ categorisation on neurodegenerative disease (Alzheimer's Disease (AD), Parkinson's Disease (PD), dementias) and mental health (e.g., depression, anxiety, stress, personality disorders) outcomes.

Establish a longitudinal cohort for the investigation of neurodegenerative diseases and mental health specifically for the LGBTQIA+ communities, designed by the communities, named by the communities and run by the communities.

**Result:**

The outcomes of this programme of work will be exponential, providing the first longitudinal cohort study of neurodegeneration and mental health which is inclusive to all LGBTQIA+ communities.

**Conclusion:**

The main, and important impact of this longitudinal study will be that for the first time, the LGBTQIA+ will be included in analyses which explore exposures and outcomes of neurodegeneration and mental health specific to these communities (e.g., marginalisation).

